# Serum Ghrelin Is Associated with Verbal Learning and Adiposity in a Sample of Healthy, Fit Older Adults

**DOI:** 10.1155/2013/202757

**Published:** 2013-07-18

**Authors:** David Bellar, Ellen L. Glickman, Lawrence W. Judge, John Gunstad

**Affiliations:** ^1^Department of Kinesiology, University of Louisiana Lafayette, 225 Cajundome Boulevard, Lafayette, LA 70504, USA; ^2^School of Health Sciences, Exercise Science Laboratory, Kent State University, Kent, OH 44243, USA; ^3^School of Physical Education, Sport and Exercise Science, Ball State University, Munice, IN 47306, USA; ^4^Department of Psychology, Kent State University, Kent, OH 44243, USA; ^5^Department of Psychiatry, Summa Health System, Akron, OH 44304, USA

## Abstract

The purpose of the present investigation was to determine the relationship between serum ghrelin concentrations, adiposity, and verbal learning in a group of healthy, fit older adults. Participants were 28 healthy older adults (age: 70.8 ± 9.3 yrs, BMI: 27.3 ± 5.7). Participants reported to the laboratory and basic anthropometric data were collected, followed by a blood draw to quantify serum ghrelin. Participants then underwent cognitive testing that included the revised Hopkins Verbal Learning Test (HVLT), as well as the Mini-Mental Status Exam (MMSE). The results of the MMSE test revealed that the volunteers were cognitively intact (MMSE 27.6 ± 1.8). A significant correlation emerged between serum ghrelin concentrations, 2 trials of the HVLT (Trial 1: *r* = 0.316, *P* = 0.05; Trial 2: *r* = 0.395, *P* = 0.03), and the sum of three-site skinfold analysis (*r* = 0.417,  *P* = 0.015). Based upon the aforementioned relationships, it appears that fasting levels of serum ghrelin are related to both verbal learning and adiposity in healthy, fit older adults.

## 1. Introduction

Cognitive functioning of older adults represents an important area of study in the United States as the lifespan of the population continues to increase with improvements in technology and modern medicine. While early work primarily focused on the pathological changes in cognitive function associated with neurological (e.g., Alzheimer's disease) or systemic medical disease (e.g., cardiovascular disease), recent studies have begun to focus on the cognitive changes found in healthy aging.

Within healthy older adults who are aerobically fit, factors such as body composition and nutritional status are known contributors to cognitive function [1]. Body fat (i.e., adiposity) in older adults who have good cardiovascular fitness, is a probable indicator of appetite. Healthy eating habits have been associated with better cognitive function in a recent large cohort study [2]. Those older adults, who are fit and have high energy stores might have higher supplies of nutritional resources, such as fatty acids and vitamins, to maintain central nervous system health while at the same time having the cardiovascular fitness to reduce their susceptibility to vascular dementia [3]. Thus in the population, of healthy and fit older adults, an increase in body fat suggesting good appetite might relate to higher levels of cognitive function. Appetite is regulated though the action of several hormones, of which ghrelin is linked to the stimulation of appetite in humans. Ghrelin expression is enhanced by caloric restriction and reduced by food intake [4]. Ghrelin arises from the cleavage of appetite regulating hormone and has receptors expressed on a variety of tissues throughout the body [4]. Ghrelin is also linked to the expression of growth hormone [5] which has been related to cognitive function in older adults [6]. 

These findings implicate the hormone ghrelin as an important factor in the cognitive changes associated with healthy aging. Ghrelin is a hormone produced by the stomach that stimulates appetite and has been shown to correlate with loss of fat-free mass in older adults [7]. In a recent study, Serra-Prat et al. [8, 9] found that in frail older adults (>75 yrs of age), levels of fasting ghrelin were lower than similar age nonfrail older adults. It was also suggested by these authors that in frail older adults, there was a loss of ghrelin prandial rhythm and that this might possibly relate to the mechanism for the anorexia associated with aging. Similarly, ghrelin has also been linked to memory performance. Diano et al. [10] reported that in an animal model, circulating ghrelin entered the hippocampus and stimulated changes that enhanced spatial learning and memory. More recent work using ghrelin receptor agonists resulted in acute increases in cognitive performance in rodents [11]. The rodents in this investigation were given two ghrelin receptor agonists and then were tested via a water maze and novel object recognition test. The rodents displayed acute improvements in performance on both tests after both ghrelin receptor agonists were administered. Recent work [12] found that ghrelin administration in rodents increases intrahippocampal nitric oxide production in a dose-dependant manner. Since nitric oxide and nitric oxide synthase activity are linked to memory formation [13] and ghrelin appears to modulate this in a dose-dependant manner, it would appear that ghrelin could have a dose-dependant relationship to performance on memory tasks. Ghrelin has been shown to cross the blood brain barrier [14]; thus, it is possible that serum ghrelin may cross the blood brain barrier and promote memory formation through dose-dependant modulation of the nitric oxide production. Thus, based upon the experimental literature, in a population of healthy fit older individuals, increased levels of ghrelin may benefit memory performance and may be related to body composition. 

Therefore, it is hypothesized that verbal learning and memory would be positively related to both levels of fasting ghrelin and adiposity in healthy, fit older adults. The purpose of the present investigation is to evaluate these relationships.

## 2. Materials and Methods

**Figure 1:**
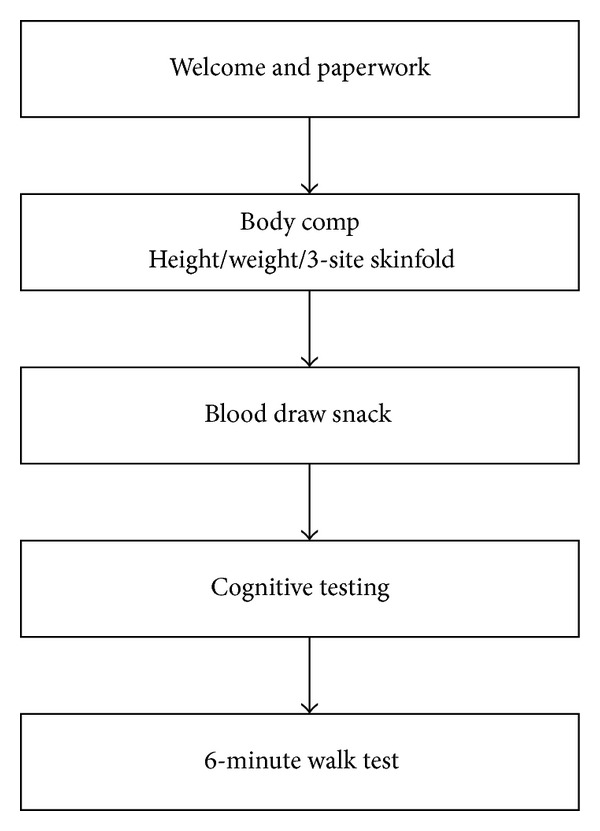
Flowchart of the protocol performed during the present investigation. Total time of participation was approximately two hours.

 The Institutional Review Board at Kent State University approved the present investigation for the use of human participants. Participants in the study were asked to report to the Applied Physiology Laboratory at Kent State University in the morning (09:00). Morning hours were selected, as there was the need for a fasting blood draw (see Figure 1). Those participants who volunteered were escorted into a laboratory room with a table and chairs and were allowed to become comfortable with their surroundings. Prior to beginning the experimental protocol, participants gave informed consent and were made aware that they were free to withdraw at any time. 

### 2.1. Body Composition Assessment

 Participants had their height measured via a stadiometer and their weight measured via a standard balance beam scale, and body mass index (BMI) was determined for each participant [15]. Each participant had three-site skinfold analysis via Lange calipers (Model 14921 Beta Technology, Santa Cruz, CA, USA) performed in order to indirectly estimate body fat. The three sites chosen included abdomen, suprailiac, and triceps. Body fat percentages were calculated according to the equations of Jackson and Pollock [16] and Jackson et al. [17]. An individual who was trained and experienced in assessing body composition via skinfolds performed the measurements.

### 2.2. Blood Collection and Analysis

After body composition assessment, participants underwent a fasted blood draw. A certified phlebotomist performed all blood draws. The phlebotomist drew via venipuncture from the antecubital space four 10 mL vials of blood in serum separator tubes with a Vacutainer needle (BD medical supplies, Franklin Lakes, NJ, USA). After collection, blood samples were spun down in a Fisher (Thermo Fisher Scientific, Rochester, NY, USA) centrifuge at 25°C for 15 min and the serum alloquated into 2 mL Nunc Cryotubes (Thermo Fisher Scientific, Rochester, NY, USA). Serum total ghrelin levels were quantified via an RIA technique (Millipore Human Ghrelin TOTAL RIA, sensitivity 100–10,000 pg/mL, Millipore Corp., Billerica, MA, USA) performed at the Cleveland Clinic Reference Laboratory (Cleveland, OH, USA). Ghrelin hormone is present in the body in both acyl (active) and deacyl (inactive) states; however, serum total ghrelin is correlated with the acyl or the active ghrelin present in the system [18, 19]. Given this correlation, the exploratory nature of the experiment, and the previous work done with similar subjects and total ghrelin [8, 19], total ghrelin was selected for analysis. At the end of the blood collection, participants were given a snack consisting of juice and a granola bar that did not exceed 300 kilocalories (8 fl oz of liquid, less than 12 grams of simple sugars). 

### 2.3. Cognitive Tests

Prior to testing, participants were given a small snack to increase their comfort and were allowed to recover from the venipuncture for 15 minutes. Though this snack may result in increased levels of circulation leptin hormone, a hormone known to relate to learning and memory, the time course for the rise in leptin concentration would make it unlikely that leptin was a mediating factor in the outcomes of the cognitive tests [20].

The first cognitive assessment administered was the Modified Mini-Mental Status Examination (MMSE). This test was administered to assess the global cognitive function of the participants. Following the MMSE, the Hopkins Verbal Learning Test-Revised [19] was administered. This instrument was administered to assess the verbal learning and the memory of the participants.

### 2.4. Cardiovascular Fitness

 This test was designed as a simple measure of cardiovascular fitness for older adults (60–94 yrs of age). The participant was asked to walk a path around the outside of a volleyball court with a perimeter of 60 yards for a total of 6 minutes. The length that the participant traveled in the minutes is recorded and compared to norms by age and gender [21]. Heart rate via a Polar heart rate monitor (Polar Electro RS800, Kempele, Finland) was recorded during this activity for safety.

### 2.5. Volunteers

Participants were 28 healthy older adults (age: 70.8 ± 9.3 yrs, BMI: 27.3 ± 5.7; see Table 1). The recruited participants ranged in age from 50 to 83 and were able to perform all the fitness measures associated with the study. The participants were not institutionalized and reported 15 ± 3 years of educational history. Exclusionary criteria included inability to perform the 6 min walk test and medication that affects cognitive function or heart rate, such as prescription beta blockers (see Table 1). 

### 2.6. Statistical Analysis

Prior to undertaking this study, a power analysis on a study with similar measures and population was undertaken to determine an adequate sample size. It was determined based upon an a priori alpha level of 0.05 that a minimum of 25 subjects needed to be recruited. Post hoc power analysis is presented along with the results. Initially, all data collected were subjected to analysis of normality. Relationships among variables were assessed via bivariate correlations. A modern statistics package (SPSS ver 16.0) was utilized to perform all calculations. Statistical significance was seta priori at an alpha level of 0.05. 

## 3. Results

 The age of the participants correlated negatively with the scores on the second and third trials of the Hopkins Verbal Learning Test (Trial 1: *r* = 0.168, achieved power = 0.221, *P* = 0.39; Trial 2: *r* = 0.378, achieved power = 0.676, *P* = 0.047; Trial 3: *r* = 0.418, achieved power = 0.766, *P* = 0.027). However, the educational experience of the participants was not related to HVLT scores (*P* > 0.43). Three significant correlations were found to the serum concentration of ghrelin. First, serum ghrelin was significantly positively correlated with the performance on Trial 1 and Trial 2 of the Hopkins Verbal Learning Test (Trial 1: *r* = 0.316, achieved power = 0.529, *P* = 0.05; Trial 2: *r* = 0.395, achieved power = 0.715, *P* = 0.03; see Table 2). A second positive correlation existed between serum ghrelin and the sum of skinfolds assessed during the body composition measurements (*r* = 0.417, achieved power = 0.763, *P* = 0.015; see Table 2). Serum ghrelin did not correlate with the performance of the participants on the 6-minute walk test (*r* = 0.018, *P* = 0.927). Serum ghrelin did not demonstrate any significant relationships to age or years of education of the participants (*r* < 0.170, *P* > 0.42). 

## 4. Discussion

The present investigation demonstrated that serum ghrelin levels were related to HVLT Trials 1 and 2 performance in a sample of healthy older adults. It should also be noted that a trend of positive correlation existed between ghrelin and HVLT Trial 3 and delayed recall (*r* = 0.295, *r* = 0.228, resp.), though these correlations were nonsignificant. Diano et al. [10] have suggested that the release of ghrelin from the hypothalamus is related to an increase in the plasticity of the hippocampus, an area of the brain that is often associated with memory. Hippocampal damage has been shown to decrease performance on tasks of verbal learning, such as the HVLT [22]. Thus, the positive association between Trial 2 performance could be indicative of a connection that exists between ghrelin and hippocampal plasticity. 

The present investigation also revealed a significant relationship between serum ghrelin and sum of skinfolds (*r* = 0.417, achieved power = 0.763, *P* = 0.015) suggesting that the volunteers with the greatest amount of adipose tissue had the highest levels of ghrelin. Though volunteers in the present investigation were in general fit from a cardiovascular prospective (as assessed by the 6-minute walk test results), the average BMI for the group places them in the in the overweight classification. It might be that the heavier (i.e., higher body weight/mass) individuals are so because they have elevated levels of ghrelin, in response to hunger. Those elevated levels could potentially increase their consumption of food, and through that consumption the heavier individuals could avoid ketogenesis and increase the consumption of vitamins. It has been reported in past research [3] that nutritional deficiencies are related to decline in cognitive function in older adults. Requejo et al. [1] reported that older adults with adequate cognitive function (MMSE ≥ 28) had greater intake of total food, fish, and alcohol. These subjects also had a greater reported intake of fatty acids, cholesterol, and vitamins. Fatty acids have been implicated in brain biochemistry as well as in cognitive function [23]. Thus, the relationships reported in the present investigation could potentially be explained as an increase in appetite and consumption that is neuroprotective, mediated by increased ghrelin responses to hunger, and demonstrated through greater sum of skinfold measurement. Recent work by Andrews et al. [24] demonstrated that peripheral ghrelin demonstrated a neuroprotective effect on the normal dopamine function in the substantia nigra pars compacta and area where dopamine is linked to the development of Parkinson's disease. Thus, some neuroprotective effects of ghrelin in the CNS have been characterized, and it is plausible that further neuroprotective effects exist. 

The present investigation is not without limitations. The sample size though large enough to establish a significant relationship warrants further investigation. In addition, a large cohort study might draw out more associations that could not be elucidated by the present investigation. Another possible limitation was the inclusion of the snack to increase the comfort of the participants; the laboratory visit was extended, and the research group decided that a small snack was warranted given the overnight fast. 

## 5. Conclusion

The present investigation has revealed a positive association between ghrelin, verbal learning, and body fat. Future work will need to elucidate the mechanisms by which these associations become manifest. It is postulated that the relationship might be due to the link between ghrelin and hippocampal plasticity as well as between ghrelin and appetite. However, these plausible explanations will need to be evaluated through further research in this area. 

## Figures and Tables

**Table 1:** Subject characteristics for males and females (M ± SD).

Variable	Males (*n* = 6)	Females (*n* = 22)	*P*-value
Age (years)	74.5 ± 7.9	69.7 ± 9.5	0.278
Height (cm)	170.0 ± 7.4	158.1 ± 6.3	0.001*
Weight (Kg)	79.3 ± 12.9	68.3 ± 15.5	0.126
Body fat (%)	26.8 ± 2.6	30.2 ± 5.1	0.126
Education (years)	14.6 ± 3.5	15.8 ± 4.0	0.528
MMSE total score	26.7 ± 1.8	27.9 ± 1.8	0.139
6-minute walk test (yards)	628.2 ± 207.2	567.9 ± 140.8	0.409

*Indicates a significant difference by gender.

**Table 2:** Bivariate correlations with serum ghrelin analysis.

Variable	Correlation with serum ghrelin	*P* value
HVLT Trial 1	*r* = 0.316	0.050*
HVLT Trial 2	*r* = 0.395	0.017*
HVLT Trial 3	*r* = 0.295	0.064
HVLT delayed recall	*r* = 0.228	0.122
Sum of skinfolds	*r* = 0.417	0.015*

Correlations are listed between the results of the revised Hopkins Verbal Learning Test (HVLT), sum of the three-site skinfold analysis (mm), and serum ghrelin concentrations. *A significant correlation.
